# Investigation of Polyester Tire Cord Glycolysis Accompanied by Rubber Crumb Devulcanization

**DOI:** 10.3390/polym14040684

**Published:** 2022-02-11

**Authors:** Kirill Kirshanov, Roman Toms, Pavel Melnikov, Alexander Gervald

**Affiliations:** M.V. Lomonosov Institute of Fine Chemical Technologies, MIREA—Russian Technological University, 119571 Moscow, Russia; kirill_kirshanov@mail.ru (K.K.); toms.roman@gmail.com (R.T.); gervald@bk.ru (A.G.)

**Keywords:** PET, polyethylene terephthalate, oligoethylene terephthalates, bis(2-hydroxyethyl) terephthalate, crumb rubber, chemical recycling, tire recycling, homogeneous glycolysis, devulcanization

## Abstract

A new method for the recycling of a polyester tire cord under the action of oligoethylene terephthalates, bis(2-hydroxyethyl) terephthalate and ethylene glycol has been proposed. The method involves simultaneous homogeneous glycolysis of polyethylene terephthalate and devulcanization of crumb rubber. Polyester cord and glycolysates were characterized by FTIR spectroscopy and gel permeation chromatography (GPC). The devulcanization process was investigated by swelling-based methods. The rate of the proposed method of homogeneous glycolysis in a melt phase was proved to be higher than one of the heterogeneous glycolysis. The assumption of a more efficient devulcanization in the presence of a softener was also confirmed. The degree of devulcanization 46.07%, the apparent degree of swelling 167.4%, and the apparent swelling rate constant 0.0902 min^−1^ were achieved. The results indicate that the proposed method made it possible to carry out the glycolysis of the polyester cord of the tire more deeply than the known heterogeneous glycolysis with various agents, but further research is needed for industrial implementation.

## 1. Introduction

Tires are one of the main sources of microplastics [1], so the recycling of end-of-life tires is an urgent task. In recent years, there has been an increasing interest in tire recycling and the most studied methods of treatment being pyrolysis and ground crumb rubber production [2]. It is known that tires are composite materials, which, in addition to rubber itself, contain other components, including textile cord. The most commonly used cord material is polyethylene terephthalate (PET) and, therefore, recycled polyester tire cord consists of PET fibers and ground tire rubber [3,4]. The reuse as milled raw material is the most developed among the recycling methods for polyester cord. For example, it can be a filler in low density polyethylene-based composites [3,4]. The recycled tire polyester cord is widely used in the production of cement and concrete, both mixed with rubber crumb [5,6] and cleaned [7,8]. The same raw material [9], or only crumb rubber [10], could be used as bitumen modifiers or reinforcement materials for expansive soils [11]. The newest application for recycled tire cord components is 3D printing using polyethylene terephthalate [12] or crumb rubber [13,14].

Polyethylene terephthalate is recycled in various ways and the most promising ones being chemolytic depolymerization methods, such as hydrolysis, acidolysis, esterolysis, alcoholysis, and glycolysis [15,16,17]. PET glycolysis is a process for the chemical recycling of polyethylene terephthalate by the action of glycols. A wide range of substances is used as glycolysis catalysts, including metal salts, complex compounds, nanoparticles, nanotubes, and ionic liquids. The common catalyst is zinc acetate. The heterogeneous glycolysis is well described in the literature, but recently homogeneous processes in solution or melt have also been proposed [18,19]. Common glycolytic agents are ethylene glycol, diethylene glycol, and 1,2-propylene glycol. Glycolysis products, such as bis(2-hydroxyethyl) terephthalate (BHET) and oligoethylene terephthalates, could also be used as agents [19].

The aforementioned recycling products are used in various fields. It is known that BHET is a plasticizer and softener for rubber, and is also used in cement composites [20]. Since PET synthesized from the purified products of glycolysis is similar to pristine PET, it can be used as preforms and fibers. Such polyethylene terephthalate can be used not only in the new polyester tire cord production, but also, for example, in medical wound dressings [21,22,23].

In recent years, the largest tires manufacturers have begun to use recycled polyester fibers: Michelin (Carbios’ enzymatic recycling technology), Continental (fibers from Oriental Industries (Suzhou) LTD), and Toyo (Ecopet Plus polyester fibers from Teijin Fibers Limited). However, it is important to note that in all these cases, post-consumer PET bottles are used instead of cord recycling. Impurities of crumb rubber and other fragments, which must either be separated or recycled along with the base material, hamper chemical recycling of polyester cord. The main process of rubber regeneration is called devulcanization [24]. It can be triggered by the number of factors, such as mechanical action and temperature [25,26,27,28], the introduction of chemical reagents, catalysts or solvents, and softeners [26,28], by the influence of bacterial enzymes [28].

The aim of the current work is to investigate the possibility of the tire cord recycling by the homogeneous glycolysis method, in which oligoesters and/or BHET are present in the reaction medium throughout the entire process. To our knowledge, this is the first time that the recycling process is carried out simultaneously with devulcanization and in a completely homogeneous mode. The substances used are both degradation agents and softeners that intensify the devulcanization of crumb rubber impurities.

## 2. Materials and Methods

### 2.1. Materials

Polyester tire cord containing 20 wt% residual rubber crumb and fragments was kindly provided by EcoSever LLC (Vologda, Russia). Ethylene glycol (EG) and diethylene glycol (DEG) were used as glycolytic agents and zinc acetate dihydrate was used as the catalyst. These reagents were purchased from Sigma Aldrich (St. Louis, MO, USA) and were used without further purification. Post-consumer transparent PET flakes with at least 95% of the main fraction, particle size from 5 to 10 mm (Tver Polymers Recycling Plant, Tver, Russia) were used for other glycolytic agents synthesis.

### 2.2. Glycolysis

Bis(2-hydroxyethyl) terephthalate and oligoethylene terephthalates are known to be PET glycolysis agents, as well as ethylene glycol and diethylene glycol [19]. The proposed novel glycolysis method is compared with known heterogeneous glycolysis at a constant total reaction time of 1.5 h and 1 wt% zinc acetate catalyst relative to the weight of polyester fiber in the raw material.

#### 2.2.1. Synthesis of Glycolytic Agents

Bis(2-hydroxyethyl) terephthalate (sample BHET-1) was synthesized as follows [19]. Then, 100 g of post-consumer PET flakes were added to a solution of 2.28 g zinc acetate dihydrate in 250 g of ethylene glycol at 190 °C, followed by stirring at 200 rpm until the disappearance of the PET phase. Then, the reaction mixture was transferred to 1 L of water and chilled for 2 h. The precipitate was filtered off and dried in an infrared dryer until constant weight was reached.

To obtain oligoethylene terephthalate (sample OET-1) [19], 19 g of PET flakes were added to 50 g of molten BHET and kept at 190 °C for 2.5 h. Thereafter, the water-soluble components (BHET, EG, zinc acetate) were extracted with water during 10 h in a Soxhlet extractor. The precipitate was dried in an infrared dryer until constant weight was reached.

#### 2.2.2. Heterogeneous Glycolysis

Heterogeneous glycolysis with ethylene glycol and diethylene glycol was carried out as follows. Polyester tire cord was added to a solution of the catalyst in the glycolysis agent at the reaction temperature (Table 1). The amount of glycolysis agent was 9 mole parts per 1 mole part of PET units, with the PET content in the cord being 80 wt%. The reaction mixture was held at a constant temperature for 1.5 h. The temperature was set below the boiling points of ethylene glycol and diethylene glycol at atmospheric pressure for Gl-1 and Gl-2, respectively.

#### 2.2.3. Novel Glycolysis

**Figure 1:**
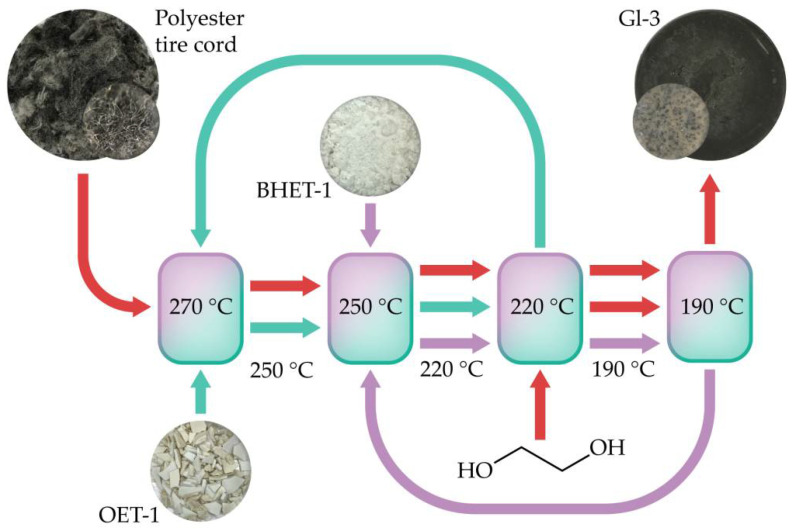
Homogeneous step-by-step glycolysis via a mixing-degradation strategy.

The novel glycolysis method is proposed based on the previous findings [19]. The process is based on the successive reduction inF the molecular weight of polyester by oligoesters. The scheme of the proposed method is shown in Figure 1.

The main advantage of the proposed glycolysis method is that the system is homogeneous at each step (co-solvent effect). The reaction rate in a homogeneous system is known to be more than an order of magnitude higher than one in a heterogeneous system. The temperatures and ratios of raw materials and glycolysis agents are shown in Figure 1 and Table 1. First, the polyester cord was mixed with the catalyst and high molecular weight oligoethylene terephthalate. This oligomer was represented by a fresh OEP-1 or its recycle flow from the subsequent stage. It should be noted that mixing must be carried out at a temperature greater than the melting point of PET. The mixing process was followed by the reaction for 30 min. The resulting stream was similarly mixed with a low molecular weight PET oligomer or monomer, sample BHET-1 or a recycle flow. The second mixing step was followed by a second chemical reaction step for 30 min. In the last step, the resulting stream was mixed with ethylene glycol, followed by a reaction for 30 min. The rubber softeners oligoethylene terephthalates or bis(2-hydroxyethyl) terephthalate are present in the reaction mixture throughout the entire process. The presence of softeners is known to speed up the devulcanization process of rubber [25,27,28]. The mixing temperatures were set above the melting points of the components so that the reaction temperature was above the melting point of the resulting mixture, i.e., took place in a homogeneous environment.

### 2.3. Cord Characterization

Modern tire cord mainly consists of the polyethylene terephthalate [4,5]. The composition of the cord used in this work was confirmed by the correlation of the FTIR spectra of mechanically purified cord and PET. The spectra were obtained by means of Spectrum 65 FT-IR spectrometer (Perkin Elmer, Waltham, MA, USA).

### 2.4. Characterization of Glycolysates

Glycolysates were separated from crumb rubber, with liquid glycolysates being filtered, and solid ones being separated mechanically.

#### 2.4.1. PET Conversion

The unreacted solid PET remaining in the reactor was isolated from the reaction mixture and weighted (m_1_) to determine the conversion. The extent of PET glycolysis (X) [18,29] was determined by Equation (1):

(1) $$X=\frac{m_{0}-m_{1}}{m_{0}},$$

where m_0_ is the initial mass of PET [26].

#### 2.4.2. Gel Permeation Chromatography (GPC)

Gel permeation chromatography (Gilson Inc., Middleton, WI, USA) with Agilent MIXED-E column, tetrahydrofuran as the mobile phase, and refractive index detector was used to determine the molecular weights of the component present in the analyzed samples. Measurements were made at the temperature of 25 °C and the flow rate of 1.0 mL/min. Narrowly dispersed polystyrene standards with M_p_ (peak molecular weight) 580; 1280; 2940; 10,110; 28,770 g/mol and polydispersity index no more than 1.12 were used for calibration (Agilent, Santa Clara, CA, USA).

#### 2.4.3. Fourier Transform Infrared Spectroscopy (FTIR)

The degree of polycondensation of oligoethylene terephthalates was determined by the ratio of bands corresponding to terminal hydroxyl groups (3350 cm^–1^) and carbonyl groups in the chain (1720 cm^–1^) [18]. FTIR spectra were obtained by means of Spectrum 65 FT-IR spectrometer (Perkin Elmer, Waltham, MA, USA).

### 2.5. Characterization of Reclaimed Rubber

The reclaimed rubber was mechanically separated from the glycolysate or polyester cord and subjected to research the methods described below.

#### 2.5.1. Degree of Devulcanization

The degree of devulcanization was determined according to ASTM D6814 and its modifications [24,27]. Then, 0.2 g of rubber from each sample was subjected to extraction with acetone in a Soxhlet extractor for 16 h and drying followed by weighting (m_i_). Then, sample was swelled in toluene during 72 h and weighted (m_t_). After that, the swollen sample was dried in an oven at 90 °C for 2 h and weighted again (m_d_).

The original rubber before devulcanization (RBD) contains soluble products, which indicates the occurrence of degradation processes in it during the previous processing. To take this fact into account, the modified equation was used to calculate the soluble fraction, and it slightly differs from that described earlier [27]:

(2) $$\%S=\frac{m_{i}-m_{d}}{m_{i}}\times 100-{\%S}_{i},$$

where S_i_ is the soluble fraction of the RBD.

The density of the rubber (ρ_d_) was evaluated by the hydrostatic weighing procedure:

(3) $$\rho_{d}={\;\rho }_{\mathrm{methanol}}\times \frac{m_{\mathrm{air}}}{m_{\mathrm{air}}-{\;m}_{\mathrm{methanol}}},$$

where m_air_ is the mass of sample weighed in air, m_methanol_ is the mass of the sample while immersed in methanol, ρ_methanol_ is the methanol density at room temperature (0.792 g/mL^3^).

The rubber volume fraction V_r_ was determined by Equation (4):

(4) $$V_{r}=\frac{m_{d}/\rho_{d}}{m_{d}/\rho_{d}+{\;m}_{s}/\rho_{s}},$$

where m_d_ is the mass of dried rubber, m_s_ = m_t_ − m_d_ is the mass of the toluene absorbed by the sample, ρ_s_ is the toluene density at room temperature (0.867 g/mL^3^).

The corrected rubber volume fraction V_rc_ was determined by Equation (5) [30]:

(5) $$V_{\mathrm{rc}}=V_{r}\times \left(1-m\times \frac{\varphi }{1-\varphi \;}\right),$$

where m = 1.7 is the Kraus interaction between the rubber and the filler, and φ = 30 vol% is the volume fraction of the filler.

The crosslink density was determined by Equation (6):

(6) $$\nu_{e}=\frac{-\left[\ln\left(1-V_{\mathrm{rc}}\right)+V_{\mathrm{rc}}+{\chi V}_{\mathrm{rc}}^{2}\right]\;}{\left[V_{1}\left(V_{\mathrm{rc}}^{1/3}-V_{\mathrm{rc}}/2\right)\right]},$$

where V_rc_ is the rubber volume fraction in the swollen sample, χ is the rubber–solvent interaction parameter (0.391), V_1_ is the molar volume of toluene (106.3 mL/mol).

The degree of devulcanization of each sample was determined by Equation (7):

(7) $$\%\mathrm{Devulc}.=\left[1-\frac{\nu_{f}}{\nu_{i}}\right]\times 100,$$

where ν_i_ and ν_f_ are, respectively, the crosslink densities of the rubber before and after devulcanization, calculated according to Equation (6).

#### 2.5.2. Horikx Plot

The Horikx theory allows one to assess whether devulcanization, degradation of the macromolecule chains, or both occur simultaneously. This theory is graphically displayed by the Horikx plot with %S vs. %Devulc. coordinates [27].

#### 2.5.3. Swelling Characterization

First, the sample was treated in the same way as during the determination of the degree of devulcanization and weighed (m_it_). Thereafter, the samples were swollen in toluene for 10, 20, 30, 40, 50, 60, 70, 80, and 90 min and weighted again (m_tt_). The apparent degree of swelling of each sample was calculated using Equation (8) [31,32]:

(8) $$\%\mathrm{Swell}.=\frac{m_{\mathrm{tt}}-m_{\mathrm{it}}}{m_{\mathrm{it}}}\times 100,$$

The apparent swelling rate constant (k) was estimated graphically in coordinates ln[(%Swell._max_ − %Swell.)/%Swell._max_] vs. time (t) according to Equation (9):

(9) $$\ln\left[\frac{\%\mathrm{Swell}._{\max}-\%\mathrm{Swell}.}{\%\mathrm{Swell}._{\max}}\right]=\mathrm{kt},$$

where %Swell._max_ is the equilibrium apparent degree of swelling.

## 3. Results and Discussion

### 3.1. Textile Cord Characterization

**Figure 2:**
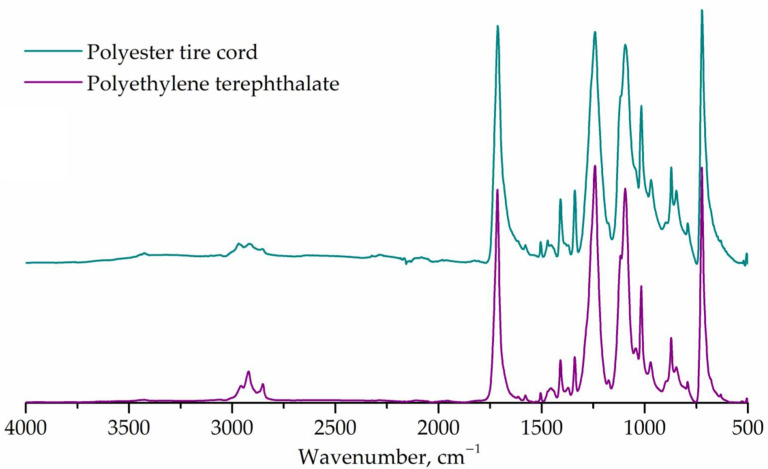
FTIR spectra of tire cord and polyethylene terephthalate.

The FTIR spectra of polyethylene terephthalate and tire cord are shown in Figure 2. The absorption bands of polyethylene terephthalate were detected at 2957, 2921, and 2851 cm^−1^ (C-H, stretching); 1715 cm^−1^ (carbonyl, stretching); 1608, 1580, and 1504 cm^−1^ (ring, stretching); 1472 and 1451 cm^−1^ (CH_2_, bending); 1409 cm^−1^ (aromatic skeleton, stretching); 1387, 1369, and 1339 cm^−1^ (CH_2_, wagging); 1243 cm^−1^ (ester group, stretching); 1176 and 1116 cm^−1^ (1,4-substituted ring); 1096 and 1042 cm^−1^ (CH_2_, deformation and C-O, vibration); 970, 897, 873, and 844 cm^−1^ (C-H, stretching); 723 cm^−1^ (C-H, out-of-plane bending). These bands correspond to known data for partially crystalline PET [33]. FTIR spectrum of tire cord shows the same bands as PET spectrum, excluding noise at 2000–2500 cm^−1^ region and weak shoulder at 1387 cm^−1^. The latter may be due to impurities or additives in secondary raw materials.

The correlation coefficient is 96%, and thus, the modern cord used in this work consists mainly of polyethylene terephthalate.

### 3.2. Obtained Glycolysis Agents Properties

Data obtained for samples BHET-1 and OET-1 are given in Supplementary Materials (Figures S1 and S2). The degree of polycondensation was determined by GPC and verified by FTIR spectroscopy. It amounted to 2.9 for OET-1 sample and 1.2 for BHET-1 sample. The number of glycolysis agent units for each of the samples was calculated from these data.

### 3.3. Textile Cord Glycolysis Investigation

**Figure 3:**
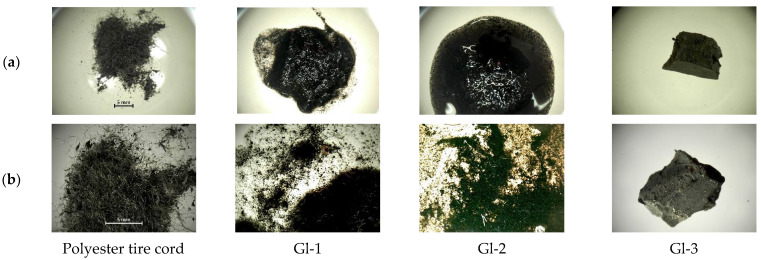
Photos of polyester tire cord and samples Gl-1, Gl-2, Gl-3: (**a**) macro; (**b**) micro.

Photographs of the original polyester tire cord and the samples obtained as a result of various processing methods are shown in Figure 3.

The resulting system, excluding rubber phase, was represented by a mixture of liquid (ethylene glycol or diethylene glycol with other substances dissolved in them) and solid (unreacted PET with other substances dissolved in it) phases.

PET conversion was determined by weighting of the solid phase (Table 2). Although the PET conversion changes from 0 and 100%, the mass of the solid phase changes from the mass of the initial polyethylene terephthalate to 0, respectively. For the proposed method of homogeneous glycolysis, the PET conversion reaches 100% already at the early stages of the process.

**Figure 4:**
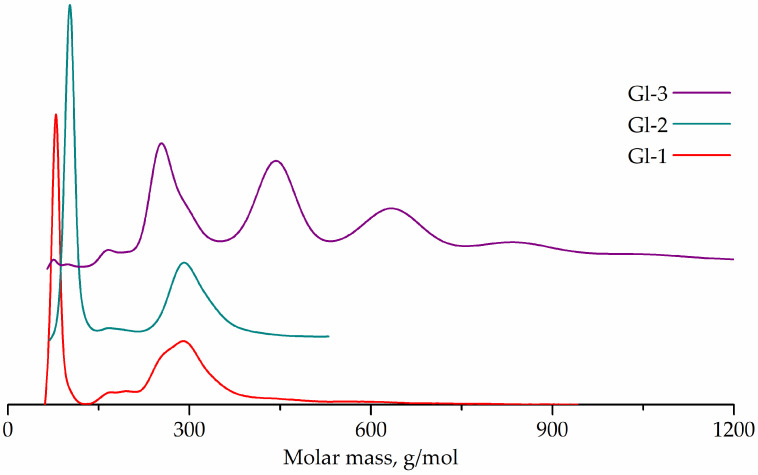
GPC curves of Gl-1, Gl-2, Gl-3 samples.

The product of glycolysis is present predominantly in the liquid phase. Since the product is not only BHET, but also oligomers, the yield of BHET itself cannot serve as a characteristic of the glycolysis depth and the latter was estimated by GPC. Liquid phase GPC curves of Gl-1 and Gl-2 samples and main phase of Gl-3 sample are shown in Figure 4.

The GPC curve of sample Gl-1 has a strong peak corresponding to unreacted ethylene glycol (68 g/mol). Additionally, weak peaks corresponding to terephthalic acid (157 g/mol) and its monoethylene glycol ester (192 g/mol), and medium peaks corresponding to terephthalic acid ethylene glycol (263 g/mol) and diethylene glycol (304 g/mol) diesters are noticeable, with these diesters being present in approximately the same amount.

Gl-2 sample was found to contain unreacted diethylene glycol (90 g/mol), terephthalic acid traces (158 g/mol) and terephthalic acid diethylene glycol diester (299 g/mol).

The GPC curve of Gl-3 sample shows the presence of unreacted ethylene glycol (68 g/mol), terephthalic acid (157 g/mol) and its monoethylene glycol ester (194 g/mol) traces, bis(2-hydroxyethyl) terephthalate (257 g/mol), as well as oligomers with degrees of polycondensation from 2 to 5 (492, 746, 1022, 1337 g/mol).

Deviations of the observed molecular weights from the calculated ones can be explained by the use of a polystyrene standard. The largest deviations are observed for compounds containing diethylene glycol or its units. Characteristics of glycolysates are given in Table 2.

The peak areas in Figure 4 correspond to the quantities of substances of the obtained products. The observed results for Gl-1 and Gl-2 confirm that the depth of glycolysis in heterogeneous glycolysis with ethylene glycol and diethylene glycol are comparable. It should also be noted that the temperature difference of 30 °C (190 °C and 220 °C for Gl-1 and Gl-2, respectively) does not affect the depth of glycolysis significantly. Hence, it could be concluded that the reaction is limited by the diffusion stage, and not by the reaction kinetics. The depth of glycolysis by the novel homogenous method surpasses heterogeneous methods, even taking into account the content of BHET and oligoesters in the glycolytic agent, with Gl-3 glycolysate having the highest number average and weight average molecular weight (Figure 4, Table 2). In addition, a larger amount of terephthalic acid was obtained during the side reaction in novel hydrolysis method than in a conventional heterogeneous process. The lower fraction of unbound ethylene glycol in the reaction mixture can explain both larger molecular weights and the yield of terephthalic acid. In heterogeneous glycolysis, the product desorbed into the liquid phase interacts with an excess of ethylene glycol or diethylene glycol. The latter also explains the formation of terephthalic acid diethylene glycol diester during heterogeneous glycolysis with ethylene glycol.

### 3.4. Devulcanization Investigation

**Figure 5:**
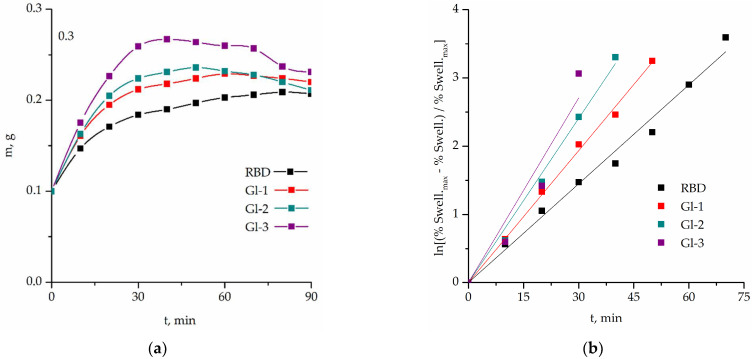
Kinetic curves of the swelling of rubber from Gl-1, Gl-2, Gl-3 in toluene at a temperature of 298.15 K (25 °C) in coordinates: (**a**) m vs. t; (**b**) ln[(%Swell._max_ − %Swell.)/%Swell._max_] vs. t, where m is the current value of gel weight.

Kinetic curves of the swelling of RBD, Gl-1, Gl-2, and Gl-3 samples are shown in Figure 5.

The apparent swelling rate constants were calculated from the slopes in Figure 5b. The characteristics of the devulcanization processes are given in Table 3. The original RBD contains soluble products, and this indicates the occurrence of degradation processes in it during the previous processing. To take this fact into account, an amendment was made to Equation (2) used to calculate the soluble fraction, and it differs slightly from that described earlier [27].

The density of the samples differs within the margin of error. The decrease in crosslink density for samples Gl-1 and Gl-2 is comparable. The lower value for Gl-2 may be due to the influence of a higher process temperature [27], since the dissolving ability of ethylene glycol and diethylene glycol with respect to carbochain macromolecules is practically the same. The greatest decrease in the crosslink density is observed for the Gl-3 sample obtained in the proposed homogeneous process, which indicates its greater devulcanization efficiency. This sample also exhibited a greater degree of swelling and a greater swelling rate constant than others and the RBD (Table 3).

**Figure 6:**
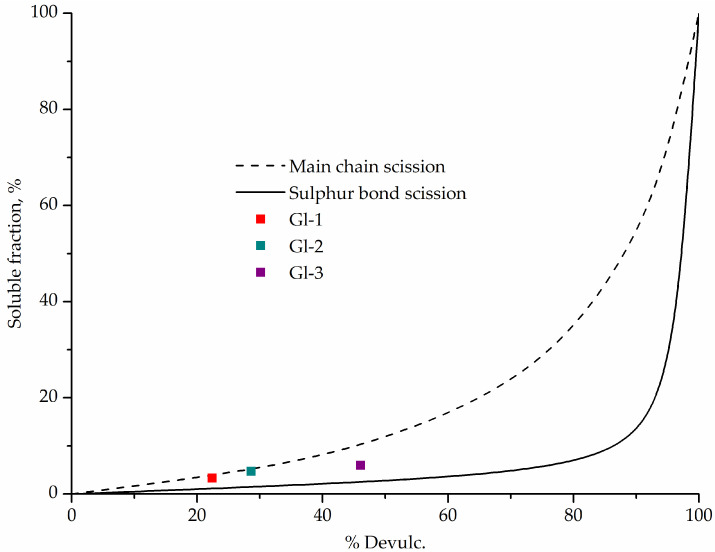
Horikx plot.

The Horikx plot was used to evaluate the relationship between crosslinks and backbone chains degradation (Figure 6). It was found that both degradation and devulcanization processes took place in all samples. A larger soluble fraction in the rubber sample obtained during the novel glycolysis process (Gl-3) is compensated by a larger decrease in crosslink density.

A smaller decrease in crosslink density than at similar temperatures and a shorter reaction time [27] can be explained by the barrier effect of glycolysate relative to oxygen, as in liquid seals. This is also confirmed by a significantly lower soluble fraction in all samples.

## 4. Conclusions

The novel method allowed one to carry out the glycolysis of the modern polyester tire cord more deeply than the known heterogeneous processes with various agents. Devulcanization proceeds simultaneously with the processes of glycolysis. Residual crumb rubber and fragments in heterogeneous glycolysis with ethylene glycol and diethylene glycol reached the degrees of devulcanization 22.39% and 28.63%, respectively. The presence of known rubber softeners bis(2-hydroxyethyl) terephthalate and oligoethylene terephthalates as glycolysis agents and the high-temperature during the process made it possible to achieve the degree of devulcanization of 46.07%. The obtained degrees of devulcanization have a good correlation with the apparent degrees of swelling and swelling rate constants.

Thus, the proposed process of simultaneous glycolysis and devulcanization is effective and is worth further investigation in order to be implemented in industry. The direction of the additional study is the variation of glycolysis agents’ ratios, temperatures, and reaction times. The main goals are the achievement of the highest yield of bis(2-hydroxyethyl) terephthalate and the highest degree of rubber devulcanization. All three components (PET, its glycolysate (BHET) and crumb rubber) are used as additives in concrete, and such a mixture can be used as is. However, questions about the prospects for using such a mixture are the subject of further research.

## Figures and Tables

**Table 1:** Conditions for obtaining samples.

Sample	Gl-1	Gl-2	Gl-3
Glycolysis method	Heterogeneous glycolysis		Novel glycolysis
Glycolytic agents	EG	DEG	OET, BHET, EG
Temperature, °C	190	220	270, 250, 220, 190
Molar ratio of agent (agent units) to PET units	9:1	9:1	3:1, 3:1, 3:1

**Table 2:** Characteristics of glycolysates Gl-1, Gl-2, and Gl-3: PET conversion, the number average (M_n_) and weight average (M_w_) molecular masses and polydispersity index (PDI).

Sample	PET Conversion, %	M_n_	M_w_	PDI
Gl-1	5	101	146	1.44
Gl-2	12	120	148	1.23
Gl-3	100	323	445	1.38

**Table 3:** Characteristics of devulcanizates.

Sample	Soluble Fraction, %	Density, g/mol	Crosslink Density, ×10^−5^ mol/cm^3^	Apparent Degree of Swelling, %	Apparent Swelling Rate Constant, min^−1^
RBD	0	1.056	3.96	108.7	0.0483
Gl-1	3.28	1.042	3.08	127.9	0.0645
Gl-2	4.71	1.037	2.83	134.0	0.0803
Gl-3	5.99	1.015	2.14	167.4	0.0902

## Data Availability

Not applicable.

## Supplementary Materials

The following supporting information can be downloaded at: https://www.mdpi.com/article/10.3390/polym14040684/s1, Figure S1: GPC curves of BHET-1, OET-1 samples, Figure S2: FTIR spectra of BHET-1, OET-1 samples.
