# Supplementary material for: Investigation of Polyester Tire Cord Glycolysis Accompanied by Rubber Crumb Devulcanization
Source: Polymers (Basel). 2022 Feb 11;14(4):684. doi: 10.3390/polym14040684 (PMC8878447; doi:10.3390/polym14040684)
Supplement: Supplementary file 1 [file polymers-14-00684-s001.zip › polymers-1583322-supplementary.pdf]

Supplementary Materials

Investigation of polyester tire cord glycolysis accompanied by rubber crumb devulcanization

Kirill Kirshanov, Roman Toms, Pavel Melnikov * and Alexander Gervald

M.V. Lomonosov Institute of Fine Chemical Technologies, MIREA—Russian Technological University, 119571 Moscow, Russia; kirill_kirshanov@mail.ru (K.K.); toms.roman@gmail.com (R.T.); melnikovsoft@mail.ru (P.M.); gervald@bk.ru (A.G.)

* Correspondence: melnikovsoft@mail.ru

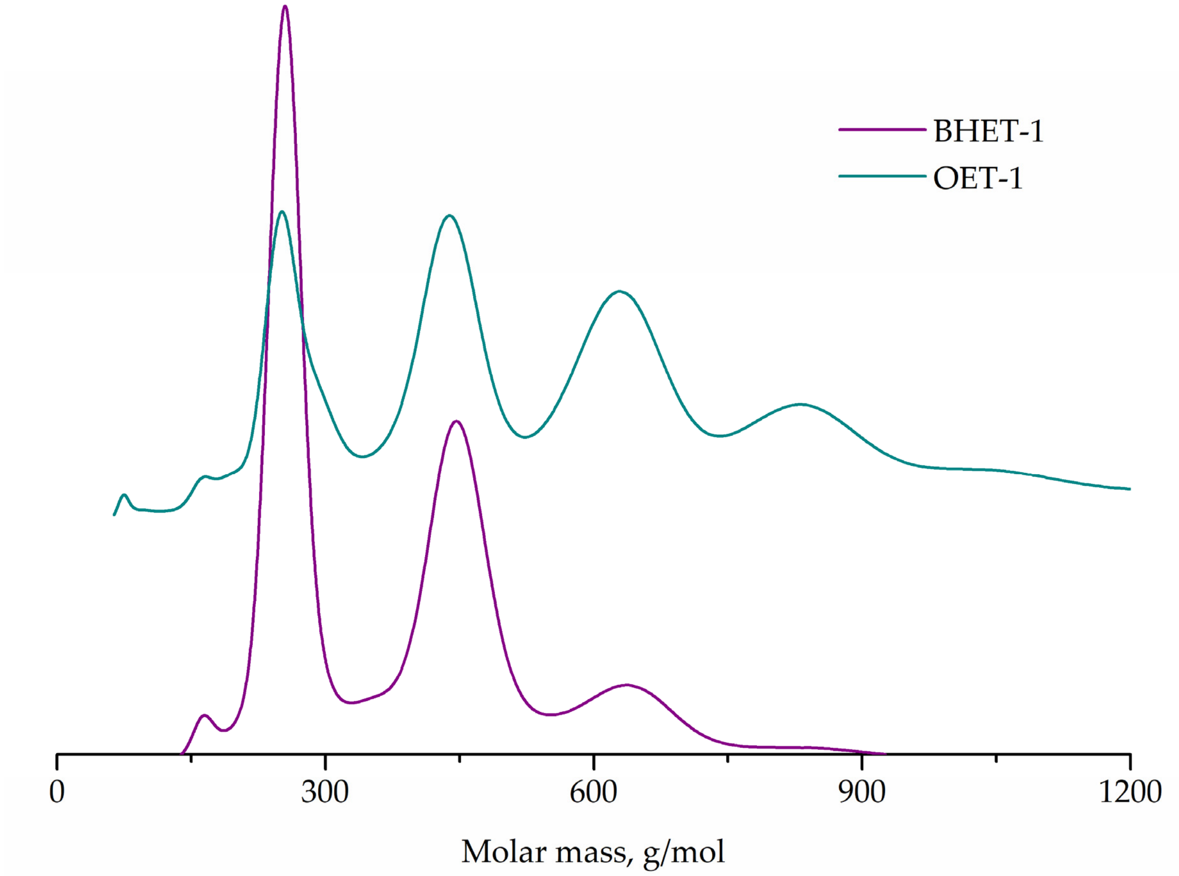

Figure S1. GPC curves of BHET-1, OET-1 samples.

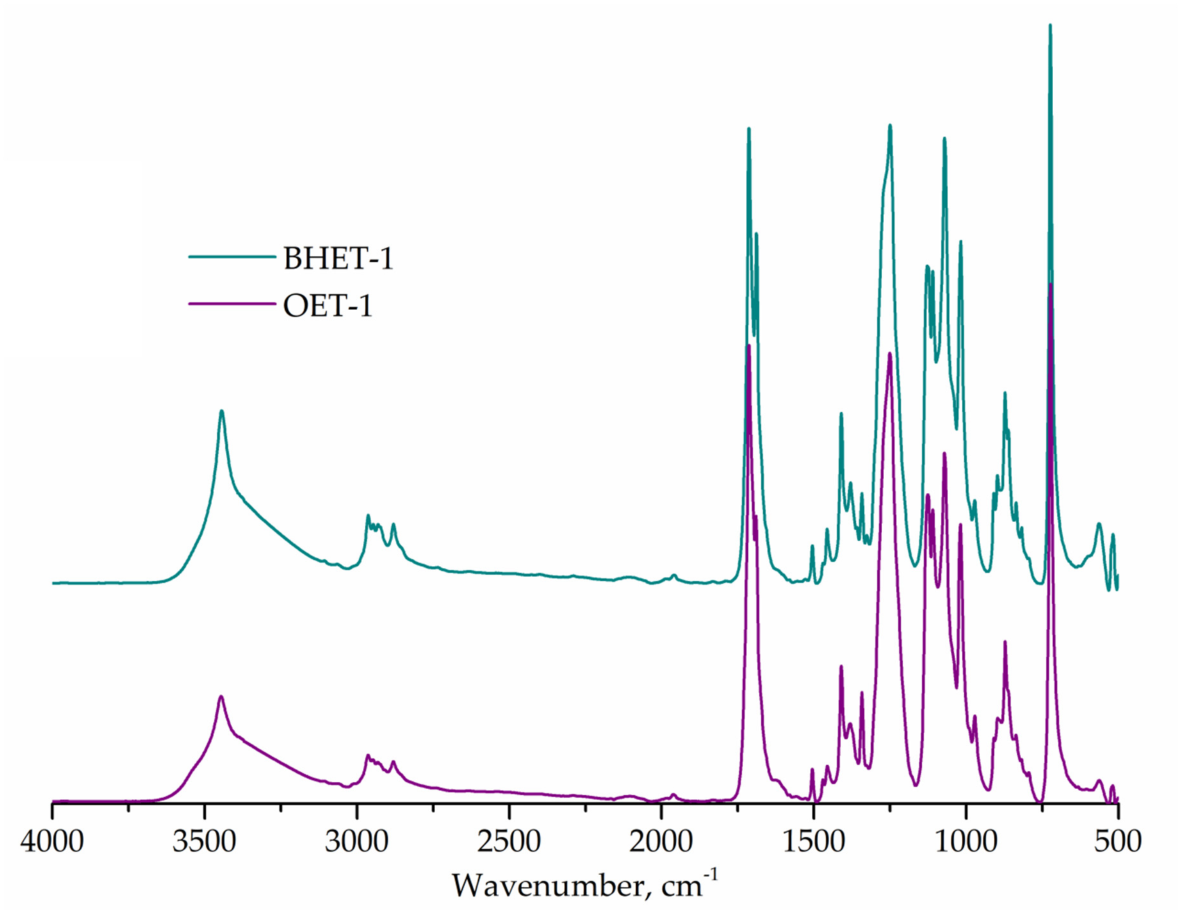

Figure S2. FTIR spectra of BHET-1, OET-1 samples.
